# Perspectives on the Barrier to Resistance for Dolutegravir + Lamivudine, a Two-Drug Antiretroviral Therapy for HIV-1 Infection

**DOI:** 10.1089/aid.2019.0171

**Published:** 2019-12-31

**Authors:** Marta Boffito, Laura Waters, Pedro Cahn, Roger Paredes, Justin Koteff, Jean Van Wyk, Tia Vincent, James Demarest, Kimberly Adkison, Romina Quercia

**Affiliations:** ^1^ Chelsea and Westminister Hospital, London, United Kingdom.; ^2^ Mortimer Market Centre, London, United Kingdom.; ^3^ Fundación Huésped, Buenos Aires, Argentina.; ^4^ Hospital Germans Trias i Pujol, Catalonia, Spain.; ^5^ ViiV Healthcare, Research Triangle Park, North Carolina.; ^6^ ViiV Healthcare, Brentford, United Kingdom.

**Keywords:** HIV, antiretroviral, genetic barrier to resistance, two-drug regimen, integrase strand transfer inhibitor

## Abstract

In HIV-1-infected patients, virological failure can occur as a consequence of the mutations that accumulate in the viral genome that allow replication to continue in the presence of antiretrovirals (ARVs). The development of treatment-emergent resistance to an ARV can limit a patient's options for future therapy, prompting the need for ARV regimens that are resilient to the emergence of resistance. The genetic barrier to resistance refers to the number of mutations in an ARV's therapeutic target that are required to confer a clinically meaningful loss of susceptibility to the drug. The emergence of resistance can be affected by pharmacological aspects of the ARV, including its structure, inhibitory quotient, therapeutic index, and pharmacokinetic characteristics. Dolutegravir (DTG) has demonstrated a high barrier to resistance, including when used in a two-drug regimen (2DR) with lamivudine (3TC). In the GEMINI-1 and GEMINI-2 studies, DTG +3TC was noninferior to DTG + emtricitabine/tenofovir disoproxil fumarate in treatment-naive participants, with similar proportions achieving HIV-1 RNA <50 copies/mL through 96 weeks. Furthermore, in the TANGO study, virological suppression was maintained at 48 weeks after switching to DTG +3TC from a tenofovir alafenamide (TAF)-based regimen compared with continuing a TAF-based regimen. Most other 2DRs with successful outcomes compared with three-drug regimens have been based on protease inhibitors (PIs); however, this class is associated with adverse metabolic effects and drug–drug interactions. In this review, we discuss the barrier to resistance in the context of a 2DR in which a boosted PI is replaced with DTG +3TC.

## Introduction

Virological failure in HIV-1-infected patients can be associated with emergence of antiretroviral (ARV) drug resistance, narrowing options for future therapy.^1^ Although the genetic barrier to resistance describes the threshold of mutations required for clinically meaningful loss of drug susceptibility,^2^ the emergence of resistance can also be influenced by the drug's structure,^3^ inhibitory quotient,^4^ therapeutic index,^5^ and pharmacokinetic forgiveness.^6^

Several two-drug regimens (2DRs) have been compared with three-drug regimens (3DRs) in clinical studies, most of which examined regimens based on boosted protease inhibitors (PIs).^7–9^ Although PIs demonstrate a high barrier to resistance,^10–12^ they are associated with metabolic adverse effects and substantial drug–drug interactions compared with other ARV classes.^13,14^ Second-generation integrase strand transfer inhibitors (INSTIs) offer the possibility of non-PI-based 2DRs. A 2DR based on the INSTI dolutegravir (DTG) demonstrated noninferiority to a 3DR in a treatment-naive population in the GEMINI-1 and GEMINI-2 studies through 48 weeks, as well as no selection of resistance mutations among those with confirmed virological withdrawal.^15^ Importantly, similar proportions of participants achieved HIV-1 RNA <50 copies/mL through 96 weeks in both study groups, with no further emergence of resistance.^16^ Furthermore, in the TANGO study, virological suppression was maintained for 48 weeks in a treatment-experienced population after switching from a tenofovir alafenamide (TAF)-based regimen to DTG + lamivudine (3TC), with no observation of confirmed virological withdrawal or treatment-emergent resistance in the DTG +3TC group.^17^ We discuss the barrier to resistance concept in the context of the 2DR DTG +3TC.

## Barrier to Resistance

ARV development focuses on optimizing new agents that are resilient to developing resistance and retain activity in the presence of existing resistance-associated mutations. Second-generation INSTIs [e.g., DTG, bictegravir (BIC)] present improved resistance profiles compared with first-generation INSTIs [e.g., raltegravir (RAL), elvitegravir (EVG)].^18–21^

In clinical trials that compared DTG and other ARVs combined with two nucleoside reverse transcriptase inhibitors (NRTIs) in treatment-naive participants, no treatment-emergent DTG resistance has been observed.^18,22–25^ Among participants in the SPRING-2 study with virological failure and available genotypic results, INSTI resistance was not observed in the DTG group (*n* = 8) but was detected in 1 (6%) of 18 in the RAL group; none of 12 in the DTG group and 4 (21%) of 19 in the RAL group developed NRTI resistance.^18^ Two studies comparing BIC- and DTG-based 3DRs reported noninferior efficacy and no treatment-emergent resistance in either group.^24,25^ The FLAMINGO and ARIA studies compared DTG and boosted PIs in treatment-naive participants with no INSTI or PI resistance observed.^22,23^

## Pharmacological Aspects

### Drug structure

The interaction between an ARV and its target at the structural level influences emergence of HIV-1 resistance. Although INSTIs have a conserved mode of binding to viral integrase,^26^ resistance mutations affect interactions between each INSTI and its binding site differently, creating varied effects on ARV activity.^3^ In a study comparing structural and functional characteristics of DTG, RAL, and EVG, DTG was shown to extend farther into the binding site and have more flexibility to adjust its position in the presence of amino acid substitutions than RAL and EVG.^3^ These characteristics are attributed to the length and flexibility of the linker connecting the tricyclic metal-chelating core and the difluorophenyl ring and may allow DTG to retain antiviral activity in the presence of mutations that confer resistance to other INSTIs.^3^

DTG exhibits a longer half-life of dissociation from wild-type integrase-DNA complex (71 h) compared with RAL (8.8 h) and EVG (2.7 h),^27^ and a dissociation half-life of 135 h has been reported for BIC.^28^ Prolonged residence time at the active site may extend the duration of efficacy, contributing to a higher barrier to resistance.^27^

### Inhibitory quotient

The inhibitory quotient expresses the potency of a drug as the ratio of drug exposure to viral susceptibility^29^ and is measured as the concentration of drug required to suppress replication of wild-type virus at a specified level, often 50% (IC_50_) or 90% (IC_90_).^10^ DTG has an IC_90_ of 0.064 μg/mL, 17-fold below plasma concentrations observed at the end of the dosing interval for a once-daily, 50-mg dose (C_trough_; 1.1 μg/mL),^30^ making DTG a potent ARV, even in the presence of mutations that reduce viral susceptibility to RAL or EVG.^31^

### Therapeutic index

To be clinically useful, an ARV should have a wide therapeutic index, meaning viral suppression can be achieved with doses well below those that cause toxicity.^5^ Because ARVs are used in patients at concentrations well above those used to evaluate susceptibility *in vitro*, prediction of the ability of a drug to suppress viral replication at clinically relevant concentrations requires understanding the dose–response relationship.^32^

DTG has a wide therapeutic index; it is able to suppress viral replication at concentrations far below those shown to cause cytotoxicity.^31^ In a pharmacokinetic study in HIV-1-infected patients, the relationship between C_trough_ and HIV-1 RNA decline for 10 days of DTG monotherapy could be described with a simple maximum effect (*E*_max_) model with *E*_max_ = −2.6 log_10_ and a 50% effective concentration of 0.036 μg/mL.^33^ In a dose-ranging study, similar proportions of participants treated with DTG at 2, 10, and 50 mg experienced drug-related adverse effects, which were mostly mild to moderate in severity.^34^ Together, these findings demonstrate that DTG is well tolerated at an effective dose, supporting a wide therapeutic index.

### Pharmacokinetic forgiveness

Pharmacokinetic forgiveness is the difference between the duration of beneficial action after dosing and the prescribed dosing interval.^35^ ARV forgiveness relates to the number of doses that can be missed without causing viral relapse. Suboptimal adherence may lead to inadequate ARV exposure, virological failure, and drug resistance.^6,36^ Forgiveness in the context of missed doses is possible when either the elimination half-life of a drug or its inhibitory effect exceeds the recommended dosing interval.^6^

DTG has a longer elimination half-life than EVG and RAL, suggesting that it may be more forgiving of missed doses.^37^ The terminal elimination half-life of DTG is ∼14 h,^30^ compared with 12.9 h for EVG when boosted with cobicistat^38^ and 10–12 h for RAL.^39^ For BIC, the half-life reported in HIV-1-infected, treatment-, or INSTI-naive participants ranged from 16 to 21 h.^40^

**FIG. 1.**
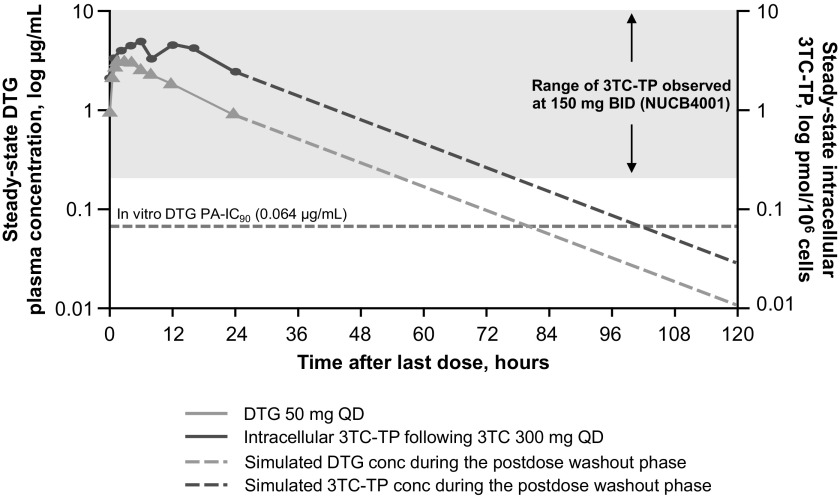
Steady-state DTG and intracellular 3TC-TP concentration-time profiles after administration of DTG 50 mg or 3TC-TP 300 mg daily.^34,41,42^ BID, twice daily; conc, concentration; DTG, dolutegravir; PA-IC_90_, protein-adjusted 90% inhibitory concentration; QD, once daily; 3TC-TP, lamivudine triphosphate.

The duration of the inhibitory effect of a drug also affects forgiveness. Plasma concentrations of DTG were >2-fold higher than the IC_90_ for 72 h after the last dose, whereas the concentration of EVG when boosted with cobicistat only exceeded the IC_95_ through 36 h, further supporting higher forgiveness of missed doses for DTG.^37^ Combination therapy should include drugs with complementary pharmacokinetic profiles, such as those demonstrated for DTG and the pharmacologically active triphosphate form of 3TC, which have similar half-lives that support once-daily dosing (Fig. 1).^34,41,42^

Synergistic interactions can occur among ARVs in combination.^43^ *In vitro* studies measuring inhibition of viral replication by DTG in combination with other ARVs found that combinations of DTG with two NRTIs [abacavir/3TC or tenofovir disoproxil fumarate/emtricitabine (TDF/FTC)] presented greater antiviral activity than expected if the effects were additive.^44^ This conclusion is consistent with a meta-analysis of studies reporting virological failure among virologically suppressed patients switched to DTG-based 2DRs, which demonstrated that the efficacy of DTG-based combination therapies was considerably higher than that of DTG monotherapy.^45^ Importantly, >50% of those experiencing virological failure on DTG monotherapy developed resistance, whereas no treatment-emergent resistance was recorded in patients who received DTG-based 2DRs.^45^

## Two-Drug Regimens

There is considerable interest in identifying 2DRs with noninferior efficacy, improved tolerability, and reduced potential for long-term adverse events compared with 3DRs.^46^ 2DRs should combine agents having high potency and a high barrier to resistance; accordingly, most successful 2DRs that have been evaluated have included boosted PIs.^7–9,47,48^ In studies investigating 2DRs, regimens composed of a ritonavir-boosted PI +3TC have shown similar outcomes to 3DR comparators and no or minimal resistance.^9,47,49^

Studies evaluating INSTI-based 2DRs include the NEAT001, PROGRESS, and SECOND-LINE studies, which all examined 2DRs composed of a ritonavir-boosted PI + RAL.^48,50,51^ In each of these studies, the 2DR demonstrated noninferior efficacy compared with the 3DR; however, mutations associated with RAL resistance emerged in participants who developed virological failure while receiving the 2DR.

DTG is a strong candidate for 2DRs based on phase III trials demonstrating its high barrier to resistance.^15,18,22,23,52^ In the GEMINI-1 and GEMINI-2 studies, virological suppression with the 2DR DTG +3TC was noninferior to that of the 3DR DTG + TDF/FTC as first-line treatment.^15^ Viral suppression (HIV-1 RNA <50 copies/mL) was achieved in 91% (655/716) and 93% (669/717) of participants in the 2DR and 3DR groups, respectively, at week 48. Both study groups exhibited similar rapid viral load log decline and median time to viral suppression overall and among participants with baseline viral load >100,000 copies/mL, including those with viral load >500,000 copies/mL at baseline; high proportions of participants achieved viral suppression, irrespective of baseline viral load.^53^ No INSTI or NRTI resistance mutations were detected in participants with confirmed virological withdrawal at 48 weeks (2DR, *n* = 6; 3DR, *n* = 4).^15^ The noninferiority of the 2DR was maintained at 96 weeks, and there was no development of resistance in either study group.^16^

In the TANGO study, participants with stable virological suppression for ≥6 months on a TAF-based regimen were randomized to switch to DTG +3TC or to continue on a TAF-based regimen.^17^ The primary endpoint was the proportion of participants with virological failure at 48 weeks according to the United States Food and Drug Administration Snapshot algorithm. The study successfully demonstrated the noninferiority of the 2DR to a TAF-based regimen, and there was no occurrence of confirmed virological withdrawal or emergence of resistance in the DTG +3TC group, making this the first randomized controlled trial in a switch population to report such findings in treatment-experienced individuals.

With promising results from GEMINI-1 and GEMINI-2 in a treatment-naive population and from TANGO in a treatment-experienced population, we are entering an era when a high resistance barrier can be achieved by combining two optimal unboosted drugs. Nonetheless, it should be noted that although these findings are encouraging, the clinical relevance of the pharmacological aspects of resistance discussed in this review is unknown. In addition, as clinical experience with the 2DR of DTG +3TC is limited to the clinical trial setting, the effect of recurrent nonadherence on the emergence of resistance remains to be determined. Finally, it is difficult to evaluate the barrier to resistance because of the small subset of participants who experienced confirmed virological withdrawal, although the low rate of confirmed virological withdrawal in the GEMINI studies and the absence of confirmed virological withdrawal in the TANGO study affirm the high efficacy of the regimen.
